# Mock theta functions and Appell–Lerch sums

**DOI:** 10.1186/s13660-018-1748-1

**Published:** 2018-07-03

**Authors:** Bin Chen

**Affiliations:** 1 School of Mathematics, Shandong University, Jinan, China; 2 School of Mathematics and Physics, Weinan Normal University, Weinan, China

**Keywords:** 11B65, 11F27, 11F03, Mock theta functions, Bilateral series, Appell–Lerch sums

## Abstract

Recently, Mortenson (Proc. Edinb. Math. Soc. 4:1–13, 2015) explored the bilateral series in terms of Appell–Lerch sums for the universal mock theta function $g_{2}{(x,q)}$. The purpose of this paper is to consider the bilateral series for the universal mock theta function $g_{3}{(x,q)}$. As a result, we present the bilateral series associated with the odd order mock theta functions in terms of Appell–Lerch sums. A very interesting congruence relationship of the bilateral series $B(\omega;q)$ for the third order mock theta function $\omega(q)$ is established. The inner relationships between the two-group bilateral series of the fifth order mock theta functions are obtained as applications.

## Introduction

In 1920, the well-known mock theta functions were first introduced by Ramanujan in his last letter to Hardy [2, 3]. Ramanujan listed seventeen functions which he called mock theta functions [4, 5]. In 2002, Zwegers [6, 7] established the relationship between mock theta functions and real analytic vector-valued modular forms. Zwegers’ breakthrough has developed the overarching theory of harmonic weak Maass forms [8–13]. These results were after Zwegers’ papers. As a result, we know that each of Ramanujan’s mock theta functions is the holomorphic part of a weight $1/2$ harmonic weak Maass form. This realization of a mock theta function has led to many applications in other associated subjects such as number theory.

Most importantly, Zagier [10] and Zwegers [6] showed that the specializations of Appell–Lerch sums are associated with mock theta functions. Recently, Hickerson and Mortenson [14, 15] have built some relations between the Hecke-type sums and Appell–Lerch sums. Furthermore, they have expressed all the mock theta functions in terms of Appell–Lerch sums.

Throughout this paper, let $q=e^{2\pi i\tau}$, $\tau\in\mathbb{H}:=\{ \tau\in\mathbf{C}|\Im(\tau)>0\}$. Suppose that *n* is a positive integer, $(a;q)_{n}$ is the *q*-Pochhammer symbol defined by

1.1 $$ (a;q)_{0}=1,\qquad (a;q)_{n}= \prod_{j=0}^{n-1} \bigl(1-aq^{j}\bigr), $$

and

1.2 $$ (a;q)_{\infty}= \prod_{j=0}^{\infty} \bigl(1-aq^{j}\bigr). $$

For one of the third order mock theta functions

1.3 $$ f(q):= 1+ \sum_{n=1}^{\infty}{ \frac{q^{n^{2}}}{(1+q)^{2}(1+q^{2})^{2} \cdots(1+q^{n})^{2}}}=\sum_{n=0}^{\infty} \frac{q^{n^{2}}}{(-q;q)^{2}_{n}}, $$

Ramanujan claimed the following.

### Ramanujan’s Claim

([5])

*As* *q* *approaches an even order* 2*k* *root of unity* *ζ* *radially within the unit disk*, *then*

1.4 $$ \lim_{q \rightarrow\zeta}{\bigl(f(q)-(-1)^{k} b(q)\bigr)= O(1)}, $$

*where the function* $b(q)$ *is defined as*

1.5 $$ \begin{aligned}[b] b(q)&:= (1-q) \bigl(1-q^{3}\bigr) \bigl(1-q^{5} \bigr)\cdots\bigl(1-2q+2q^{4}-\cdots\bigr) \\ &= \bigl(q;q^{2}\bigr)_{\infty}\sum _{n=-\infty}^{\infty}{(-1)^{n}q^{n^{2}}}= \frac {(q;q)_{\infty}}{(-q;q)^{2}_{\infty}}.\end{aligned} $$

We point that $b(q)$ is a modular form of weight $1/2$ with respect to *τ*.

Folsom, Ono, and Rhoades [16, 17] obtained two closed formulas for the implied constant $O(1)$ in two different ways. As a result, they pointed out that Ramanujan’s claim is the special case of their theorem. The author and Zhou [18] established an inner relationship of the two theorems of Folsom–Ono–Rhoades.

Bajpai et al. [19] showed that some of the bilateral series of mock theta functions have played the role of $b(q)$ in Ramanujan’s claim. Interesting enough, these associated bilateral series are modular forms on some congruence group $\Gamma_{1}(N)$, where the associated bilateral series of mock theta functions is defined as follows.

Let $M(q):= \sum_{n\geq0}{c(n;q)}$ be a mock theta function, its associated bilateral series is defined as

1.6 $$ B(M;q):=\sum_{n\in\mathbb{Z}}c(n;q). $$

Meanwhile, let *n* be a positive integer. Using the substitution $n \rightarrow-n$ in [20] of $(a;q)_{n}$, we have

1.7 $$ (a;q)_{-n}:=\frac{(-a)^{-n}q^{{n(n+1)}/2}}{(a^{-1}q;q)_{n}}. $$

Recall that the Appell–Lerch sums are defined by [14, 15]

1.8 $$ m(x,q,z):=\frac{1}{j(z;q)}\sum_{r=-\infty}^{\infty} \frac {(-1)^{r}q^{r(r-1)/2}z^{r}}{1-q^{r-1}xz}, $$

where

1.9 $$ j(x;q):=(x)_{\infty}(q/x)_{\infty}(q)_{\infty}=\sum _{n=-\infty}^{\infty }{(-1)^{n}q^{n(n-1)/2}x^{n}}. $$

After that, the author and Zhou [21] found that the bilateral series $B(f;q)$ of the third order mock theta function $f(q)$ is a mixed mock modular form of weight $1/2$. And then we expressed it in terms of Appell–Lerch sums. In fact, we got the following.

### Theorem 2.2 of [21]

*Let* $B(f;q)$ *be the bilateral series of third order Ramanujan’s mock theta function* $f(q)$. *Then*

1.10 $$ H_{2}(q)=4\sum_{n \in\mathbb{Z}}q^{n+1}(-q)_{n}^{2}=B(f;q)= \sum_{n \in \mathbb{Z}}\frac{q^{n^{2}}}{(-q)_{n}^{2}} $$

*is a mixed mock modular form of weight* $1/2$. *Moreover*, *let* $\tilde{H}_{2}(q)=(-q)_{\infty}^{-2}H_{2}(q)$, *then* $\tilde{H}_{2}(q)$ *is a mock theta function*, *where*

1.11 $$ H_{2}(q)=\sum_{n=-\infty}^{-1} \frac{q^{n^{2}}}{(-q)_{n}^{2}}+4\sum_{n=-\infty}^{-1}q^{n+1}(-q)_{n}^{2}. $$

### Corollary 2.6 of [21]

*In the notation above*, *we see that*

1.12 $$ H_{2}(q)=B(f;q)=\sum_{n \in\mathbb{Z}} \frac {q^{n^{2}}}{(-q)_{n}^{2}}=4(-q)_{\infty}^{2} m(1,q,-1) $$

*is a mixed mock modular form of weight* $1/2$.

We know that the two important universal mock theta functions [13] are defined as

$$ g_{2}(x,q):=\sum_{n=0}^{\infty}{ \frac {q^{n(n+1)/2}(-q;q)_{n}}{(x;q)_{n+1}(x^{-1}q;q)_{n+1}}} $$

and

$$ g_{3}(x,q):=x^{-1} \Biggl(-1+\sum _{n=0}^{\infty}{\frac {q^{n^{2}}}{(x;q)_{n+1}(x^{-1}q;q)_{n}}} \Biggr). $$

Mortenson [1] obtained Ramanujan’s radial limits of *q*-series and some even order mock theta functions by constructing the bilateral series of the universal mock theta function $g_{2}{(x,q)}$ in terms of Appell–Lerch sums.

He first defined the function

1.13 $$ S_{1}(\omega;q):=\bigl(1+\omega^{-1}\bigr)\sum _{n=0}^{\infty}{\frac {(q;q^{2})_{n}(-1)^{n}q^{(n+1)^{2}}}{(-wq;q^{2})_{n+1}(-q/\omega;q^{2})_{n+1}}}. $$

Then, for *a* and *m* integers with *m* positive, he defined

1.14 $$ J_{a,m}:=j\bigl(q^{a};q^{m}\bigr),\qquad J_{m}:=J_{m,3m}=\prod_{i\geq1} \bigl(1-q^{mi}\bigr),\qquad \overline{J}_{a,m}:=j \bigl(-q^{a};q^{m}\bigr). $$

Mortenson proved the following results.

### Theorem 5.1 of [1]

*If* *ζ* *is a primitive even order* 2*k* *root of unity*, *k* *is odd*, *as* *q* *approaches* *ζ* *radially within the unit disk*, *then*

1.15 $$ \lim_{q\rightarrow\zeta}\biggl(S_{1}(1;q)+ \frac{J_{1,2}^{2}}{\bar {J}_{0,1}} \biggr)=-2\sum_{n=0}^{(k-1)/2}\frac{{\zeta^{2n+1}}(-\zeta;\zeta ^{2})_{n}^{2}}{(\zeta;\zeta^{2})_{n+1}}. $$

### Corollary 6.2 of [1]

*The bilateral series of the universal mock theta function* $g_{2}{(x,q)}$ *is*

1.16 $$\begin{aligned} B(g_{2};x,q)&:=\sum_{n=0}^{\infty}{ \frac {q^{n(n+1)/2}(-q;q)_{n}}{(x;q)_{n+1}(x^{-1}q;q)_{n+1}}}+\sum_{n=0}^{\infty} \frac{1}{2}\frac{q^{n}(q/x;q)_{n}(x;q)_{n}}{(-q;q)_{n}} \end{aligned}$$

1.17 $$\begin{aligned} &=-\frac{j(x;q)}{2J_{2}}g_{3}(-x;q)+\frac {J_{2}^{3}}{J_{1,2}j(x^{2};q^{2})}+ \frac{1}{2x}\frac {J_{2}^{10}j(-x^{2};q^{2})}{J_{1}^{4}J_{4}^{4}j(x^{2};q^{2})j(-qx^{2};q^{2})} \end{aligned}$$

1.18 $$\begin{aligned} &\quad{}-\frac{1}{2x}\frac{J_{2,4}^{2}j(x;q)}{j(-x;q)j(-qx^{2};q^{2})}. \end{aligned}$$

As an application of Corollary 6.2, he obtained the following.

### Corollary 6.3 of [1]

*If* *ζ* *is a primitive odd order* $2k+1$ *root of unity*, *k* *is odd*, *then*, *as* *q* *approaches* *ζ* *radially within the unit disk*, *we get*

1.19 $$ \lim_{q\rightarrow\zeta} \biggl(B_{2}(q)-\frac{J_{4}^{5}}{J_{2}^{4}}- \frac {1}{4}q^{-1}\frac{J_{4}^{17}}{J_{2}^{8}J_{8}^{8}}+\frac{1}{4}q^{-1} \frac {J_{4}J_{1}^{4}}{J_{2}^{2}J_{8}^{2}} \biggr) =-\frac{1}{2}\sum_{n=0}^{k} \frac{{\zeta^{2n}}(\zeta;\zeta ^{2})_{n}^{2}}{(-\zeta^{2};\zeta^{2})_{n}}, $$

*where* $B_{2}(q)$ *is the second order mock theta function defined by*

1.20 $$ B_{2}(q):=\sum_{n=0}^{\infty} \frac {q^{n}(-q;q^{2})_{n}}{(q;q^{2})_{n+1}}=g_{2}\bigl(q,q^{2}\bigr). $$

In view of *q*-hypergeometric relations between universal mock theta functions and Appell–Lerch sums, considering the substitution $n\rightarrow-n$ in the tail of the bilateral series of the universal mock theta function $g_{3}{(x,q)}$, we present the bilateral series of the universal mock theta function $g_{3}{(x,q)}$ in terms of Appell–Lerch sums. Furthermore, we express the associated bilateral series of odd order mock theta functions in terms of Appell–Lerch sums. As an application, the associated Ramanujan radial limits of these mock theta functions can be constructed as well. Surprisingly, by relating with the new result of Chan and Mao [22], we get a very interesting congruence relationship of the bilateral series $B(\omega ;q)$ for the third order mock theta function $\omega(q)$.

## Preliminaries

First, it is well known that the Appell–Lerch sums satisfy several functional equations and identities [1] such as the following.

For generic $x, z, z_{0}, z_{1}\in\mathbb{C}^{*}$, then

2.1 $$\begin{aligned}& m(x,q,z)=m(x,q,qz), \end{aligned}$$

2.2 $$\begin{aligned}& m(x,q,z)=x^{-1}m\bigl(x^{-1},q,z^{-1} \bigr), \end{aligned}$$

2.3 $$\begin{aligned}& m(qx,q,z)=1-xm(x,q,z), \end{aligned}$$

2.4 $$\begin{aligned}& m(x,q,z_{1})-m(x,q,z_{0})=\frac {z_{0}J_{1}^{3}j(z_{1}/z_{0};q)j(xz_{0}z_{1};q)}{j(z_{0};q)j(z_{1};q)j(xz_{0};q)j(xz_{1};q)}, \end{aligned}$$

2.5 $$\begin{aligned}& m(x,q,z)=m\bigl(x,q,x^{-1}z^{-1}\bigr). \end{aligned}$$

According to the results in [14, 21], we can get the following relationship.

### Lemma 2.1

*For* $a,b \neq0$, *we have*

2.6 $$\begin{aligned}[b] &\sum_{n=0}^{\infty}{ \frac {a^{-n-1}b^{-n}q^{n^{2}}}{(-a^{-1};q)_{n+1}(-qb^{-1};q)_{n}}}+\sum_{n=1}^{\infty}(-a q;q)_{n-1}(-b;q)_{n}q^{n} \\ &\quad=\frac{(-a q)_{\infty}}{b(q)_{\infty}(-qb^{-1})_{\infty}}j(-b;q)m(a/b,q,-b).\end{aligned} $$

### Lemma 2.2

*For the universal mock theta function* $g_{3}(x,q)$, *the bilateral series* $B(g_{3};x,q)$ *in terms of Appell–Lerch sums is*

2.7 $$ B(g_{3};x,q)=-\frac{1}{x}+\frac{1}{x^{3}} \frac{1}{(q)_{\infty }}j(x,q)m\bigl(x^{-2},q,x\bigr). $$

### Proof

Substituting $a=-x^{-1}$, $b=-x$ in the identity of Lemma 2.1, we have

2.8 $$\begin{aligned}[b] -x\sum_{n=-\infty}^{\infty}\frac{q^{n^{2}}}{(x)_{n+1}(q/x)_{n}}&=-x \Biggl(\sum_{n=0}^{\infty}\frac{q^{n^{2}}}{(x)_{n+1}(q/x)_{n}}+\sum _{n=-\infty }^{-1}\frac{q^{n^{2}}}{(x)_{n+1}(q/x)_{n}} \Biggr) \\ &=-\frac{1}{x}\frac{1}{(q)_{\infty}}j(x;q)m\bigl(x^{-2},q,x\bigr).\end{aligned} $$

Namely, we get

2.9 $$ \begin{aligned}[b] &{-}\frac{1}{x}+\frac{1}{x}\sum _{n=0}^{\infty}\frac {q^{n^{2}}}{(x)_{n+1}(q/x)_{n}}-\frac{1}{x^{2}}\sum _{n=0}^{\infty }(q/x)_{n}(x)_{n+1}q^{n+1} \\ &\quad=-\frac{1}{x}+\frac{1}{x^{3}}\frac{1}{(q)_{\infty}}j(x;q)m \bigl(x^{-2},q,x\bigr).\end{aligned} $$

Combining with the definition of $g_{3}(x,q)$, we get

2.10 $$ B(g_{3};x,q)=-\frac{1}{x}+\frac{1}{x^{3}} \frac{1}{(q)_{\infty }}j(x;q)m\bigl(x^{-2},q,x\bigr). $$

 □

## Bilateral series of odd order mock theta functions

### Theorem 3.1

*For the third order mock theta functions* [3, 14, 23],

3.1 $$\begin{aligned}& f(q)=\sum_{n=0}^{\infty}{ \frac {q^{n^{2}}}{(-q;q)^{2}_{n}}}=2-2g_{3}(-1,q), \end{aligned}$$

3.2 $$\begin{aligned}& \phi(q)=\sum_{n=0}^{\infty}{ \frac {q^{n^{2}}}{(-q^{2};q^{2})_{n}}}=(1-i) \bigl(1+ig_{3}(i,q)\bigr) \end{aligned}$$

3.3 $$\begin{aligned}& \psi(q)=\sum_{n=1}^{\infty} \frac {q^{n^{2}}}{(q;q^{2})_{n}}=qg_{3}\bigl(q,q^{4}\bigr), \end{aligned}$$

3.4 $$\begin{aligned}& \chi(q)=\sum_{n=0}^{\infty} \frac {q^{n^{2}}(-q)_{n}}{(-q^{3};q^{3})_{n}}=(1+\omega) \bigl(1-\omega g_{3}(-\omega,q)\bigr), \end{aligned}$$

3.5 $$\begin{aligned}& \omega(q)=\sum_{n=0}^{\infty}{ \frac {q^{2n(n+1)}}{(q;q^{2})^{2}_{n+1}}}=g_{3}\bigl(q,q^{2}\bigr), \end{aligned}$$

3.6 $$\begin{aligned}& \nu(q)=\sum_{n=0}^{\infty}{ \frac {q^{n(n+1)}}{(-q;q^{2})_{n+1}}}=g_{3}(i\sqrt{q},q), \end{aligned}$$

3.7 $$\begin{aligned}& \rho(q)=\sum_{n=0}^{\infty} \frac {q^{2n(n+1)}(q;q^{2})_{n+1}}{(q^{3};q^{6})_{n+1}}=g_{3}\bigl(\omega q,q^{2}\bigr), \end{aligned}$$

*where* *ω* *denotes a primitive cube root of unity*.

*Then the bilateral series in terms of Appell–Lerch sums of them are the following*, *respectively*:

3.8 $$\begin{aligned}& B(f;q)= 4(-q;q)_{\infty}^{2} m(1,q,-1), \end{aligned}$$

3.9 $$\begin{aligned}& B(\phi;q)=2i\bigl(-q^{2};q^{2}\bigr)_{\infty} m(-1,q,i), \end{aligned}$$

3.10 $$\begin{aligned}& B(\psi;q)= -1+\bigl(q;q^{4}\bigr)_{\infty} \bigl(q^{3};q^{4}\bigr)_{\infty} m\bigl(q^{2},q^{4},q^{3} \bigr)=-1+\bigl(q;q^{2}\bigr)_{\infty} m\bigl(q^{2},q^{4},q^{3} \bigr), \end{aligned}$$

3.11 $$\begin{aligned}& B(\chi;q)= \omega(1+\omega) (-\omega;q)_{\infty}\bigl(- \omega^{2} q;q\bigr)_{\infty } m(\omega,q,-\omega), \end{aligned}$$

3.12 $$\begin{aligned}& B(\omega;q)= \frac{1}{q} \bigl(-1+\bigl(q^{2};q^{2} \bigr)_{\infty} m\bigl(q^{2},q^{2},q\bigr) \bigr), \end{aligned}$$

3.13 $$\begin{aligned}& B(\nu;q)= q^{-\frac{1}{2}} \bigl(1-i\bigl(-q;q^{2} \bigr)_{\infty} m\bigl(-q,q,-iq^{\frac {1}{2}}\bigr) \bigr), \end{aligned}$$

3.14 $$\begin{aligned}& B(\rho;q)= \frac{\omega^{2}}{q} \bigl(-1+\bigl(\omega q;q^{2} \bigr)_{\infty}\bigl(\omega ^{2} q;q^{2} \bigr)_{\infty} m\bigl(\omega^{2} q^{2},q^{2}, \omega^{2} q\bigr) \bigr). \end{aligned}$$

### Proof

By using Lemma 2.2 and identity (1.9), we deduce the bilateral series of third order mock theta functions as follows, respectively:

3.15 $$\begin{aligned}& B(f;q)= 2-2B(g_{3};-1,q) \\& \phantom{B(f;q)}= 2-2 \biggl(1-\frac{1}{(q)_{\infty}}j(-1,q)m(1,q,-1) \biggr) \\& \phantom{B(f;q)}= \frac{2}{(q)_{\infty}}(-1)_{\infty}(-q)_{\infty}(q)_{\infty }m(1,q,-1) \\& \phantom{B(f;q)}= 4(-q)_{\infty}^{2} m(1,q,-1), \end{aligned}$$

3.16 $$\begin{aligned}& B(\phi;q)=(1-i) \bigl(1+iB(g_{3};i,q) \bigr) \\& \phantom{B(\phi;q)}=(1-i) \biggl[1+i\biggl(-\frac{1}{i}+\frac{1}{i^{3}} \frac{1}{(q)_{\infty }}\biggr)j(i,q)m(-1,q,i) \biggr] \\& \phantom{B(\phi;q)}=(i-1)\frac{1}{(q)_{\infty}}(i)_{\infty}(q/i)_{\infty}(q)_{\infty }m(-1,q,i) \\& \phantom{B(\phi;q)}=(i-1) (i)_{\infty}(-iq)_{\infty}m(-1,q,i) \\& \phantom{B(\phi;q)}= (i-1) (1-i) (iq)_{\infty}(-iq)_{\infty}m(-1,q,i) \\& \phantom{B(\phi;q)}= 2i \bigl(-q^{2};q^{2}\bigr)_{\infty} m(-1,q,i), \end{aligned}$$

where the identity $(a;q)_{\infty}(-a;q)_{\infty}=(a^{2};q^{2})_{\infty}$ is used.

3.17 $$ \begin{aligned}[b] B(\psi;q)&= q B\bigl(g_{3};q,q^{4}\bigr) \\ &=q \biggl(-\frac{1}{q}+\frac{1}{q^{3}}\frac{1}{(q^{4};q^{4})_{\infty }}j \bigl(q,q^{4}\bigr)m\bigl(q^{-2},q^{4},q\bigr) \biggr) \\ &=-1+\frac{1}{(q^{4};q^{4})_{\infty}}\bigl(q;q^{4}\bigr)_{\infty} \bigl(q^{3};q^{4}\bigr)_{\infty }\bigl(q^{4};q^{4} \bigr)_{\infty}q^{-2}m\bigl(q^{-2},q^{4},q \bigr) \\ &= -1+ \bigl(q;q^{4}\bigr)_{\infty} \bigl(q^{3};q^{4}\bigr)_{\infty}m\bigl(q^{2},q^{4},q^{-1} \bigr) \\ &=-1+ \bigl(q;q^{4}\bigr)_{\infty} \bigl(q^{3};q^{4}\bigr)_{\infty}m\bigl(q^{2},q^{4},q^{3} \bigr) \\ &=-1+ \bigl(q;q^{2}\bigr)_{\infty}m\bigl(q^{2},q^{4},q^{3} \bigr),\end{aligned} $$

where identities (2.1) and (2.2) are used.

3.18 $$\begin{aligned}& \begin{aligned}[b] B(\chi;q)&= (1+\omega) \bigl(1-\omega B(g_{3};-\omega,q) \bigr) \\ &= (1+\omega) \biggl[1-\omega \biggl(\frac{1}{\omega}- \frac{1}{\omega ^{3}}\frac{1}{(q)_{\infty}}j(-\omega,q)m\bigl(\omega^{-2},q,- \omega\bigr) \biggr) \biggr] \\ &=(1+\omega)\frac{\omega}{(q)_{\infty}}j(-\omega,q)m(\omega,q,-\omega ) \\ &=(1+\omega)\frac{\omega}{(q)_{\infty}} (-\omega)_{\infty}(q,-\omega )_{\infty}(q)_{\infty}m(\omega,q,-\omega) \\ &=\omega(1+\omega) (-\omega)_{\infty}\bigl(-\omega^{2} q \bigr)_{\infty}m(\omega ,q,-\omega),\end{aligned} \end{aligned}$$

3.19 $$\begin{aligned}& \begin{aligned}[b] B(\omega;q)&= B\bigl(g_{3};q,q^{2}\bigr) \\ &= -\frac{1}{q}+\frac{1}{q}\frac{1}{(q^{2};q^{2})_{\infty }}q^{-2}j \bigl(q,q^{2}\bigr)m\bigl(q^{-2},q^{2},q\bigr) \\ &= \frac{1}{q} \biggl(-1+\frac{1}{(q^{2};q^{2})_{\infty}} \bigl(q;q^{2}\bigr)_{\infty }^{2}\bigl(q^{2};q^{2} \bigr)_{\infty}m\bigl(q^{2},q^{2},q^{-1} \bigr) \biggr) \\ &= \frac{1}{q} \bigl(-1+\bigl(q;q^{2}\bigr)_{\infty}^{2} m\bigl(q^{2},q^{2},q\bigr) \bigr),\end{aligned} \end{aligned}$$

3.20 $$\begin{aligned}& \begin{aligned}[b] B(\nu;q)&= B(g_{3};i\sqrt{q},q) \\ &= \frac{1}{\sqrt{q}}+\frac{1}{i^{3} q^{3/2}}\frac{1}{(q)_{\infty }}j{(i \sqrt{q},q)}m\bigl(-q^{-1},q,i\sqrt{q}\bigr) \\ &= q^{-\frac{1}{2}} +iq^{-3/2}\frac{1}{(q)_{\infty}}(i \sqrt{q})_{\infty }(-i\sqrt{q})_{\infty}(q)_{\infty}m \bigl(-q^{-1},q,i\sqrt{q}\bigr) \\ &= q^{-\frac{1}{2}} +iq^{-1/2}\bigl(-q;q^{2} \bigr)_{\infty}q^{-1}m\bigl(-q^{-1},q,i\sqrt {q}\bigr) \\ &=q^{-\frac{1}{2}} \bigl(1-i\bigl(-q;q^{2}\bigr)_{\infty}m \bigl(-q,q,-iq^{\frac {1}{2}}\bigr) \bigr),\end{aligned} \end{aligned}$$

where the identities $(a;q)_{\infty}(-a;q)_{\infty}=(a^{2};q^{2})_{\infty }$, (2.1, and (2.2) are used.

3.21 $$ \begin{aligned}[b] B(\rho;q)&= B\bigl(g_{3};\omega q,q^{2}\bigr) \\ &= -\frac{1}{\omega q}+\frac{1}{q^{3}}\frac{1}{(q^{2};q^{2})_{\infty }}j\bigl( \omega q,q^{2}\bigr)m\bigl(\omega q^{-2},q^{2}, \omega q\bigr) \\ &= -\frac{\omega^{2}}{q}+\frac{1}{q^{3}}\frac{1}{(q^{2};q^{2})_{\infty }}\bigl( \omega q;q^{2}\bigr)_{\infty}\bigl(\omega^{2} q;q^{2}\bigr)_{\infty}\bigl(q^{2};q^{2} \bigr)_{\infty }m\bigl(\omega q^{-2},q^{2},\omega q\bigr) \\ &=\frac{\omega^{2}}{q} \bigl(-1+\bigl(\omega q;q^{2} \bigr)_{\infty}\bigl(\omega^{2} q;q^{2} \bigr)_{\infty}m\bigl(\omega^{2} q^{2},q^{2}, \omega^{2} q^{-1}\bigr) \bigr) \\ &=\frac{\omega^{2}}{q} \bigl(-1+\bigl(\omega q;q^{2} \bigr)_{\infty}\bigl(\omega^{2} q;q^{2} \bigr)_{\infty}m\bigl(\omega^{2} q^{2},q^{2}, \omega^{2} q\bigr) \bigr),\end{aligned} $$

where identities (2.1) and (2.2) are used. □

### Remark

Similarly, by making use of Lemma 2.2 and identity (1.9), as well as using formulas (2.1) and (2.2), the bilateral series of the fifth and seventh order mock theta functions can be obtained by carefully computing as well.

### Theorem 3.2

*For the fifth order mock theta functions* [3, 14, 23, 24],

3.22 $$\begin{aligned}& f_{0}(q)=\sum_{n=0}^{\infty} \frac{q^{n^{2}}}{(-q)_{n}}=\frac {J_{5,10}J_{2,5}}{J_{1}}-2q^{2}g_{3} \bigl(q^{2},q^{10}\bigr), \end{aligned}$$

3.23 $$\begin{aligned}& \phi_{0}(q)=\sum_{n=0}^{\infty }{q^{n^{2}} \bigl(-q;q^{2}\bigr)_{n}}=qg_{3} \bigl(-q,-q^{5}\bigr)+\frac {J_{10}j(-q^{2};-q^{5})}{J_{2,10}}, \end{aligned}$$

3.24 $$\begin{aligned}& \psi_{0}(q)=\sum_{n=0}^{\infty }{q^{(n+2)(n+1)/2}(-q)_{n}}=q^{2}g_{3} \bigl(q^{2},q^{10}\bigr)+\frac {qJ_{5}J_{1,10}}{J_{2,5}}, \end{aligned}$$

3.25 $$\begin{aligned}& F_{0}(q)=\sum_{n=0}^{\infty} \frac {q^{2n^{2}}}{(q;q^{2})_{n}}=1+qg_{3}\bigl(q,q^{5}\bigr)- \frac{qJ_{10}\bar {J}_{5,20}}{J_{4,10}}, \end{aligned}$$

3.26 $$\begin{aligned}& \chi_{0}(q)=\sum_{n=0}^{\infty} \frac {q^{n}}{(q^{n+1})_{n}}=2+3qg_{3}\bigl(q,q^{5}\bigr)- \frac{J_{5}^{2} J_{2,5}}{J_{1,5}^{2}}, \end{aligned}$$

3.27 $$\begin{aligned}& f_{1}(q)=\sum_{n=0}^{\infty} \frac{q^{n(n+1)}}{(-q)_{n}}=\frac {J_{5,10}J_{1,5}}{J_{1}}-2q^{3}g_{3} \bigl(q^{4},q^{10}\bigr), \end{aligned}$$

3.28 $$\begin{aligned}& \phi_{1}(q)=\sum_{n=0}^{\infty}{q^{(n+1)^{2}} \bigl(-q;q^{2}\bigr)_{n}}=q^{2} g_{3} \bigl(q^{2},-q^{5}\bigr)+\frac{qJ_{10}j(q;-q^{5})}{J_{4,10}}, \end{aligned}$$

3.29 $$\begin{aligned}& \psi_{1}(q)=\sum_{n=0}^{\infty}{q^{n(n+1)/2}(-q)_{n}}=q^{3} g_{3}\bigl(q^{4},q^{10}\bigr)+\frac{J_{5}J_{3,10}}{J_{1,5}}, \end{aligned}$$

3.30 $$\begin{aligned}& F_{1}(q)=\sum_{n=0}^{\infty} \frac {q^{2n(n+1)}}{(q;q^{2})_{n+1}}=qg_{3}\bigl(q^{2},q^{5}\bigr)+ \frac{J_{10}\bar {J}_{5,20}}{J_{2,10}}, \end{aligned}$$

3.31 $$\begin{aligned}& \chi_{1}(q)=\sum_{n=0}^{\infty}{ \frac {q^{n}}{(q^{n+1};q)_{n+1}}}=3qg_{3}\bigl(q^{2},q^{5}\bigr)+ \frac{J_{5}^{2} J_{1,5}}{J_{2,5}^{2}}, \end{aligned}$$

3.32 $$\begin{aligned}& \Phi(q)=-1+\sum_{n=0}^{\infty} \frac {q^{5n^{2}}}{(q;q^{5})_{n+1}(q^{4};q^{5})_{n}}=qg_{3}\bigl(q,q^{5}\bigr), \end{aligned}$$

3.33 $$\begin{aligned}& \Psi(q)=-1+\sum_{n=0}^{\infty} \frac {q^{5n^{2}}}{(q^{2};q^{5})_{n+1}(q^{3};q^{5})_{n}}=q^{2} g_{3}\bigl(q^{2},q^{5} \bigr), \end{aligned}$$

*where* *ω* *denotes a primitive cube root of unity*.

*Then the bilateral series in terms of Appell–Lerch sums of these associated functions are the following*, *respectively*:

3.34 $$\begin{aligned}& B(f_{0};q)= 2-2\bigl(q^{2};q^{10} \bigr)_{\infty}\bigl(q^{8};q^{10}\bigr)_{\infty} m \bigl(q^{4}, q^{10}, q^{8}\bigr)+\frac{J_{5,10}J_{2,5}}{J_{1}}, \end{aligned}$$

3.35 $$\begin{aligned}& B(\phi_{0};q)= 1-\bigl(-q;-q^{5}\bigr)_{\infty} \bigl(q^{4};-q^{5}\bigr)_{\infty} m\bigl(q^{2},-q^{5},q^{4} \bigr)+\frac{J_{10}j(-q^{2};-q^{5})}{J_{2,10}}, \end{aligned}$$

3.36 $$\begin{aligned}& B(\psi_{0};q)= -1+\bigl(q^{2};q^{10} \bigr)_{\infty}\bigl(q^{8};q^{10}\bigr)_{\infty} m \bigl(q^{4},q^{10},q^{8}\bigr)+\frac{qJ_{5}J_{1,10}}{J_{2,5}}, \end{aligned}$$

3.37 $$\begin{aligned}& B(F_{0};q)= \bigl(q;q^{5}\bigr)_{\infty} \bigl(q^{4};q^{5}\bigr)_{\infty} m\bigl(q^{2},q^{5},q^{4} \bigr)-\frac {qJ_{10}\bar{J}_{5,20}}{J_{4,10}}, \end{aligned}$$

3.38 $$\begin{aligned}& B(\chi_{0};q)= -1+ 3\bigl(q;q^{5}\bigr)_{\infty} \bigl(q^{4};q^{5}\bigr)_{\infty} m\bigl(q^{2},q^{5},q^{4} \bigr)-\frac{J_{5}^{2} J_{2,5}}{J_{1,5}^{2}}, \end{aligned}$$

3.39 $$\begin{aligned}& B(f_{1};q)= \frac{2}{q} \bigl(1-\bigl(q^{4};q^{10} \bigr)_{\infty }\bigl(q^{6};q^{10}\bigr)_{\infty} m\bigl(q^{8},q^{10},q^{6}\bigr) \bigr)+ \frac {J_{5,10}J_{1,5}}{J_{1}}, \end{aligned}$$

3.40 $$\begin{aligned}& B(\phi_{1};q)= -1+\bigl(q^{2};-q^{5} \bigr)_{\infty}\bigl(-q^{3};-q^{5}\bigr)_{\infty} m\bigl(q^{4},-q^{5},-q^{3}\bigr)+ \frac{qJ_{10}j(q;-q^{5})}{J_{4,10}}, \end{aligned}$$

3.41 $$\begin{aligned}& B(\psi_{1};q)= \frac{1}{q} \bigl(-1+\bigl(q^{4};q^{10} \bigr)_{\infty }\bigl(q^{6};q^{10}\bigr)_{\infty} m\bigl(q^{8},q^{10},q^{6}\bigr) \bigr)+ \frac {J_{5}J_{3,10}}{J_{1,5}}, \end{aligned}$$

3.42 $$\begin{aligned}& B(F_{1};q)= \frac{1}{q} \bigl(-1+\bigl(q^{2};q^{5} \bigr)_{\infty}\bigl(q^{3};q^{5}\bigr)_{\infty} m \bigl(q^{4},q^{5},q^{3}\bigr) \bigr)+ \frac{J_{10}\bar{J}_{5,20}}{J_{2,10}}, \end{aligned}$$

3.43 $$\begin{aligned}& B(\chi_{1};q)= \frac{3}{q} \bigl(-1+\bigl(q^{2};q^{5} \bigr)_{\infty}\bigl(q^{3};q^{5}\bigr)_{\infty } m \bigl(q^{4},q^{5},q^{3}\bigr) \bigr)+ \frac{J_{5}^{2} J_{1,5}}{J_{2,5}^{2}}, \end{aligned}$$

3.44 $$\begin{aligned}& B(\Phi;q)=-1+\bigl(q;q^{5}\bigr)_{\infty}\bigl(q^{4};q^{5} \bigr)_{\infty} m\bigl(q^{2},q^{5},q^{4} \bigr), \end{aligned}$$

3.45 $$\begin{aligned}& B(\Psi;q)= -1+\bigl(q^{2};q^{5}\bigr)_{\infty} \bigl(q^{3};q^{5}\bigr)_{\infty} m\bigl(q^{4},q^{5},q^{3} \bigr). \end{aligned}$$

### Theorem 3.3

*For the seventh order mock theta functions* [3, 14, 23, 24],

3.46 $$\begin{aligned}& \mathcal{F}_{0}(q)=\sum_{n=0}^{\infty}{ \frac {q^{n^{2}}}{(q^{n+1};q)_{n}}}=2+2qg_{3}\bigl(q,q^{7}\bigr)- \frac{J_{3,7}^{2}}{J_{1}}, \end{aligned}$$

3.47 $$\begin{aligned}& \mathcal{F}_{1}(q)=\sum_{n=1}^{\infty}{ \frac {q^{n^{2}}}{(q^{n};q)_{n}}}=2q^{2} g_{3}\bigl(q^{2},q^{7} \bigr)+\frac {qJ_{1,7}^{2}}{J_{1}}, \end{aligned}$$

3.48 $$\begin{aligned}& \mathcal{F}_{2}(q)=\sum_{n=0}^{\infty}{ \frac {q^{n(n+1)}}{(q^{n+1};q)_{n+1}}}=2q^{2} g_{3}\bigl(q^{3},q^{7} \bigr)+\frac{J_{2,7}^{2}}{J_{1}}. \end{aligned}$$

*Then the bilateral series in terms of Appell–Lerch sums of these functions are the following*, *respectively*:

3.49 $$\begin{aligned}& B(\mathcal{F}_{0};q)= 2\bigl(q;q^{7} \bigr)_{\infty}\bigl(q^{6};q^{7}\bigr)_{\infty} m\bigl(q^{2},q^{7},q^{6}\bigr)- \frac{J_{3,7}^{2}}{J_{1}}, \end{aligned}$$

3.50 $$\begin{aligned}& B(\mathcal{F}_{1};q)= -2+2\bigl(q^{2};q^{7} \bigr)_{\infty}\bigl(q^{5};q^{7}\bigr)_{\infty} m \bigl(q^{4},q^{7},q^{5}\bigr)+\frac{qJ_{1,7}^{2}}{J_{1}}, \end{aligned}$$

3.51 $$\begin{aligned}& B(\mathcal{F}_{1};q)= \frac{2}{q} \bigl(-1+ \bigl(q^{3};q^{7}\bigr)_{\infty }\bigl(q^{4};q^{7} \bigr)_{\infty} m\bigl(q^{6},q^{7},q^{4} \bigr) \bigr)+\frac{J_{2,7}^{2}}{J_{1}}. \end{aligned}$$

### Corollary 3.4

*After carefully studying the bilateral series of the fifth order mock theta functions in Theorem *3.2, *it is not difficult to find that these bilateral series can be divided into two categories as follows*:

1. $B(f_{0};q)$, $B(\phi_{0};q)$, $B(\psi_{0};q)$, $B(F_{0};q)$, $B(\chi _{0};q)$, *and* $B(\Phi;q)$;
2. $B(f_{1};q)$, $B(\phi_{1};q)$, $B(\psi_{1};q)$, $B(F_{1};q)$, $B(\chi _{1};q)$, *and* $B(\Psi;q)$.

### Remark

We point out that each bilateral series of the fifth order mock theta functions in the same category can be represented by each other.

### Corollary 3.5

*For the fifth order mock theta functions and their associated bilateral series*, *we have*

3.52 $$\begin{aligned}& B(f_{0};q)+B(\psi_{0};q)=1+\frac{J_{5,10}J_{2,5}}{J_{1}}+ \frac {qJ_{5}J_{1,10}}{J_{2,5}}, \end{aligned}$$

3.53 $$\begin{aligned}& B(f_{0};q)-B\bigl(\Phi;q^{2}\bigr)=1+\frac{J_{5,10}J_{2,5}}{J_{1}}, \end{aligned}$$

3.54 $$\begin{aligned}& B(f_{0};q)-B\bigl(\phi_{0};-q^{2}\bigr)=1+ \frac{J_{5,10}J_{2,5}}{J_{1}}-\frac {J_{20}\bar{J}_{4,10}}{J_{4,20}}, \end{aligned}$$

3.55 $$\begin{aligned}& B(f_{0};q)+B\bigl(F_{0};q^{2}\bigr)=2+ \frac{J_{5,10}J_{2,5}}{J_{1}}-\frac {q^{2}J_{20}\bar{J}_{10,40}}{J_{8,20}}, \end{aligned}$$

3.56 $$\begin{aligned}& 3B(f_{0};q)+B\bigl(\chi_{0};q^{2}\bigr)=5+ \frac{3J_{5,10}J_{2,5}}{J_{1}}-\frac {J_{10}^{2} J_{4,10}}{J_{2,10}^{2}}, \end{aligned}$$

3.57 $$\begin{aligned}& B(\phi_{0};-q)+B(F_{0};q)=1+\frac{J_{10}\bar{J}_{2,5}}{J_{2,10}}- \frac {qJ_{10}\bar{J}_{5,20}}{J_{4,10}}, \end{aligned}$$

3.58 $$\begin{aligned}& B(\phi_{0};-q)+B(\Phi;q)=\frac{J_{10}j(-q^{2};q^{5})}{J_{2,10}}=\frac {J_{10}\bar{J}_{2,5}}{J_{2,10}}, \end{aligned}$$

3.59 $$\begin{aligned}& 3B(\phi_{0};-q)+B(\chi_{0};q)=2+\frac{3J_{10}\bar {J}_{2,5}}{J_{2,10}}- \frac{J_{5}^{2} J_{2,5}}{J_{1,5}^{2}}, \end{aligned}$$

3.60 $$\begin{aligned}& B(\psi_{0};q)+B\bigl(\phi_{0};-q^{2}\bigr)= \frac{qJ_{5}J_{1,10}}{J_{2,5}}+\frac {J_{20}\bar{J}_{4,10}}{J_{4,20}}, \end{aligned}$$

3.61 $$\begin{aligned}& B(\psi_{0};q)-B\bigl(\Phi;q^{2}\bigr)=\frac{qJ_{5}J_{1,10}}{J_{2,5}}, \end{aligned}$$

3.62 $$\begin{aligned}& B(\psi_{0};q)-B\bigl(F_{0};q^{2}\bigr)=-1+ \frac{q^{2}J_{20}\bar {J}_{10,40}}{J_{8,20}}+\frac{qJ_{5}J_{1,10}}{J_{2,5}}, \end{aligned}$$

3.63 $$\begin{aligned}& 3B(\psi_{0};q)-B\bigl(\chi_{0};q^{2}\bigr)=-2+ \frac{J_{10}^{2} J_{4,10}}{J_{2,10}^{2}}+\frac{3J_{5,10}J_{2,5}}{J_{1}}, \end{aligned}$$

3.64 $$\begin{aligned}& B(F_{0};q)-B(\Phi;q)=1-\frac{qJ_{10}\bar{J}_{5,20}}{J_{4,10}}, \end{aligned}$$

3.65 $$\begin{aligned}& 3B(F_{0};q)-B(\chi_{0};q)=1+\frac{J_{5}^{2} J_{2,5}}{J_{1,5}^{2}}- \frac {3qJ_{10}\bar{J}_{5,20}}{J_{4,10}}, \end{aligned}$$

3.66 $$\begin{aligned}& B(\chi_{0};q)-3B(\Phi;q)=2-\frac{J_{5}^{2} J_{2,5}}{J_{1,5}^{2}}, \end{aligned}$$

*and*

3.67 $$\begin{aligned}& qB(f_{1};q)+2B\bigl(\phi_{1};-q^{2}\bigr) = \frac{qJ_{5,10}J_{1,5}}{J_{1}}-\frac {q^{2}J_{20}\bar{J}_{2,10}}{J_{8,20}}, \end{aligned}$$

3.68 $$\begin{aligned}& B(f_{1};q)+2B(\psi_{1};q)= \frac{J_{5,10}J_{1,5}}{J_{1}}+ \frac {J_{5}J_{3,10}}{J_{1,5}}, \end{aligned}$$

3.69 $$\begin{aligned}& B(f_{1};q)+2qB\bigl(F_{1};q^{2}\bigr)= \frac{J_{5,10}J_{1,5}}{J_{1}}+\frac {2qJ_{20}\bar{J}_{10,40}}{J_{4,20}}, \end{aligned}$$

3.70 $$\begin{aligned}& 3B(f_{1};q)+2qB\bigl(\chi_{1};q^{2}\bigr)= \frac{3J_{5,10}J_{1,5}}{J_{1}}+\frac {2qJ_{10}^{2} J_{2,10}}{J_{4,10}^{2}}, \end{aligned}$$

3.71 $$\begin{aligned}& qB(f_{1};q)+2B\bigl(\Psi;q^{2}\bigr)=\frac{qJ_{5,10}J_{1,5}}{J_{1}}, \end{aligned}$$

3.72 $$\begin{aligned}& qB(\psi_{1};q)-B\bigl(\phi_{1};-q^{2}\bigr)= \frac{q^{2}J_{20}\bar {J}_{2,10}}{J_{8,20}}+\frac{qJ_{5}J_{3,10}}{J_{1,5}}, \end{aligned}$$

3.73 $$\begin{aligned}& qB\bigl(F_{1};q^{2}\bigr)-B(\phi_{1};-q)= \frac{qJ_{10}\bar {J}_{1,5}}{J_{4,10}}+\frac{qJ_{10}\bar{J}_{5,20}}{J_{2,10}}, \end{aligned}$$

3.74 $$\begin{aligned}& qB(\chi_{1};-q)-3B(\phi_{1};q)= \frac{3qJ_{10}\bar {J}_{1,5}}{J_{4,10}}+ \frac{qJ_{5}^{2} J_{1,5}}{J_{2,5}^{2}}, \end{aligned}$$

3.75 $$\begin{aligned}& B(\Psi;q)-B(\phi_{1};q)= \frac{qJ_{10}\bar{J}_{1,5}}{J_{4,10}}, \end{aligned}$$

3.76 $$\begin{aligned}& B(\psi_{1};q)-qB\bigl(F_{1};q^{2}\bigr)= \frac{J_{5}J_{3,10}}{J_{1,5}}-\frac {qJ_{20}\bar{J}_{10,40}}{J_{4,20}}, \end{aligned}$$

3.77 $$\begin{aligned}& 3B(\psi_{1};q)-qB\bigl(\chi_{1};q^{2}\bigr)= \frac{3J_{5}J_{3,10}}{J_{1,5}}-\frac {qJ_{10}^{2} J_{2,10}}{J_{4,10}^{2}}, \end{aligned}$$

3.78 $$\begin{aligned}& qB(\psi_{1};q)-B\bigl(\Psi;q^{2}\bigr)= \frac{qJ_{5}J_{3,10}}{J_{1,5}}, \end{aligned}$$

3.79 $$\begin{aligned}& 3B(F_{1};q)-B(\chi_{1};q)=\frac{3J_{10}\bar{J}_{5,20}}{J_{2,10}}- \frac {J_{5}^{2} J_{1,5}}{J_{2,5}^{2}}, \end{aligned}$$

3.80 $$\begin{aligned}& qB(F_{1};q)-B(\Psi;q)= \frac{qJ_{10}\bar{J}_{5,20}}{J_{2,10}}, \end{aligned}$$

3.81 $$\begin{aligned}& qB(\chi_{1};q)-3B(\Psi;q)=\frac{qJ_{5}^{2} J_{1,5}}{J_{2,5}^{2}}. \end{aligned}$$

### Remark

Similar relationships of the bilateral series for the third and seventh order mock theta functions are mysterious.

## An application of the congruence relationship on the bilateral series

In 2012, Chan and Mao [22] obtained two congruences for an infinite family of Appell–Lerch sums. In order to introduce their results, we first give the following definition.

For any integers *m*, *j*, and *p* satisfying $1 \leq j \leq p-1$, define the integer $a_{m,j,p}$ such that

4.1 $$ \begin{aligned}[b] \sum_{n=0}^{\infty}a_{m,j,p}(n)q^{n}:&=-m \bigl(q^{mp},q^{p},q^{j}\bigr) \\ &=\frac{1}{(q^{j},q^{p-j},q^{p};q^{p})_{\infty}}\sum_{n=-\infty }^{\infty} \frac{(-1)^{n}q^{pn(n+1)/2+jn+j}}{1-q^{pn+pm+j}},\end{aligned} $$

where $(x_{1},x_{2},\ldots,x_{m};q)_{\infty}:=(x_{1};q)_{\infty}\cdots(x_{m};q)_{\infty}$.

Then they proved the following result.

### Theorem 1.3 of [22]

*For any integer* *m* *and any two coprime integers* *p* *and* *j* *such that* $p \geq2$ *and* $1 \leq j \leq p-1$, *we obtain*

4.2 $$ \sum_{n=0}^{\infty}a_{m,j,p} \bigl(pn+pj-j^{2}\bigr)q^{n}=(-1)^{m}p \frac {q^{m(m-1)/2}(q^{p};q^{p})_{\infty}^{4}}{(q;q^{3})_{\infty }^{3}(q^{j},q^{p-j};q^{p})_{\infty}^{2}}, $$

*and for any integer* *m* *and any two coprime integers* 2*p* *and* *j* *such that* $p \geq1$ *and* $1 \leq j \leq2p-1$, *we have*

4.3 $$ \sum_{n=0}^{\infty}a_{m,j,p}(2pn+p)q^{n}=(-1)^{m}p \frac {q^{m(m-1)/2+(j-1)/2}(-q^{j},-q^{p-j};q^{p})_{\infty }(q^{2p};q^{2p})_{\infty}^{4}}{(q;q^{3})_{\infty }^{3}(q^{j},q^{2p-j};q^{2p})_{\infty}^{2}(-q^{p};q^{p})_{\infty}^{2}}. $$

By considering these results, we can construct the congruence relationship on the bilateral series $B(\omega;q)$ of the third order mock theta function $\omega(q)$. Then we get a very interesting result as follows.

### Theorem 4.1

*Let* $B(\omega;q)$ *be the bilateral series of the third order mock theta function* $\omega(q)$, *and define*

4.4 $$ \frac{qB(\omega;q)+1}{(q^{2};q^{2})_{\infty}}:=\sum_{n=0}^{\infty}c(n)q^{n}, $$

*then we have*

4.5 $$ a_{1,1,2}(2n+1)\equiv c(2n+1) \pmod{2}, $$

*where* $a_{m,j,p}(n)$ *is defined in* (4.1).

### Proof

Considering the bilateral series $B(\omega;q)$ in Theorem 3.1, we have

4.6 $$ B(\omega;q)=\frac{1}{q} \bigl(-1+\bigl(q^{2};q^{2} \bigr)_{\infty} m\bigl(q^{2},q^{2},q\bigr) \bigr). $$

Thus, we get

4.7 $$ \frac{qB(\omega;q)+1}{(q^{2};q^{2})_{\infty}}= m\bigl(q^{2},q^{2},q\bigr). $$

Considering the definition of $a_{m,j,p}(n)$, we can get

4.8 $$ \sum_{n=0}^{\infty}a_{1,1,2}(n)q^{n}=-m \bigl(q^{2},q^{2},q\bigr), $$

then we have

4.9 $$ \sum_{n=0}^{\infty}c(n)q^{n}= \frac{qB(\omega;q)+1}{(q^{2};q^{2})_{\infty}}= m\bigl(q^{2},q^{2},q\bigr)=-\sum _{n=0}^{\infty}a_{1,1,2}(n)q^{n}. $$

By using the result of Chan and Mao [22], we have

4.10 $$ \sum_{n=0}^{\infty}a_{1,1,2}(2n+1)q^{n}=-2 \frac{(q^{2};q^{2})_{\infty }^{4}}{(q;q^{3})_{\infty}^{3}(q,q;q^{2})_{\infty}^{2}}. $$

Then we get

4.11 $$ \sum_{n=0}^{\infty}c(2n+1)q^{n}=- \sum_{n=0}^{\infty }a_{1,1,2}(2n+1)q^{n}=2 \frac{(q^{2};q^{2})_{\infty}^{4}}{(q;q^{3})_{\infty }^{3}(q,q;q^{2})_{\infty}^{2}}. $$

 □
